# Pilomatrix carcinoma of the scalp with pulmonary metastasis: A case report of a complete response to oral endoxan and etoposide

**DOI:** 10.3892/ol.2014.2021

**Published:** 2014-04-01

**Authors:** DENIZ ARSLAN, ŞEYDA GÜNDÜZ, FATMA AVCI, ALPARSLAN MERDIN, ALI MURAT TATLI, MÜKREMIN UYSAL, DENIZ TURAL, CUMHUR İBRAHIM BAŞSORGUN, BURHAN SAVAŞ

**Affiliations:** 1 Department of Medical Oncology, Akdeniz University Hospital, Konyaalti, Antalya, Turkey; 2 Department of Internal Medicine, Akdeniz University Hospital, Konyaalti, Antalya, Turkey; 3 Department of Medical Oncology, Afyon Kocatepe University, Ahmet Necdet Sezer Research and Practice Hospital, Afyon, Turkey; 4 Department of Pathology, Akdeniz University Hospital, Konyaalti, Antalya, Turkey

**Keywords:** pilomatrix carcinoma, pulmonary metastasis, cyclophosphamide, etoposide

## Abstract

Pilomatrix carcinoma is an extremely rare skin tumor derived from basaloid cells in the hair follicles; it often exhibits locally aggressive behavior with a tendency toward local recurrence. The average age of occurrence is 45 years, and there appears to be a male to female incidence ratio of 4:1. Although pilomatrix carcinomas are predominantly identified in the neck and scalp, there are studies in the literature reporting other tumor development sites, including the upper extremities, torso and popliteal fossa. If diagnosed at an early stage, this malignant tumor is generally treated with wide surgical resection. However, for the advanced-stage tumors, there are no standard treatment procedures known to produce good results. The current study presents the case of a 76-year-old male with pilomatrix carcinoma originating from the scalp with metastases to the lung. The patient had a rapid and complete clinical response following an oral combination chemotherapy regimen of cyclophosphamide and etoposide.

## Introduction

Pilomatrix carcinoma is a locally aggressive tumor, usually occurring on the head and neck. A literature review revealed that the mean age of diagnosis with pilomatrix carcinoma is 45 years, and it occurs more often in middle-aged males, with a male to female ratio of 4:1 (1). In numerous patients diagnosed with pilomatrix carcinoma, a direct invasion to the adjacent bone has been observed (2,3). Local recurrence is expected if the tumor has not been excised with a wide surgical margin. However, despite its limited metastatic potential, the tumor may occasionally present rapid progression and widespread metastases. Aggressive surgical treatment consisting of wide local excision is indicated for pilomatrix carcinoma and the goal of the surgery is to obtain negative surgical margins, however, wide surgical excision is not an adequate intervention. Adjuvant radiotherapy may be administered following surgery (4). In addition, patients with local invasion or metastatic properties may be treated with chemotherapy and radiotherapy (5). The current study reports a rare case of pilomatrix carcinoma. The patient provided written informed consent.

## Case report

**Figure 1:**
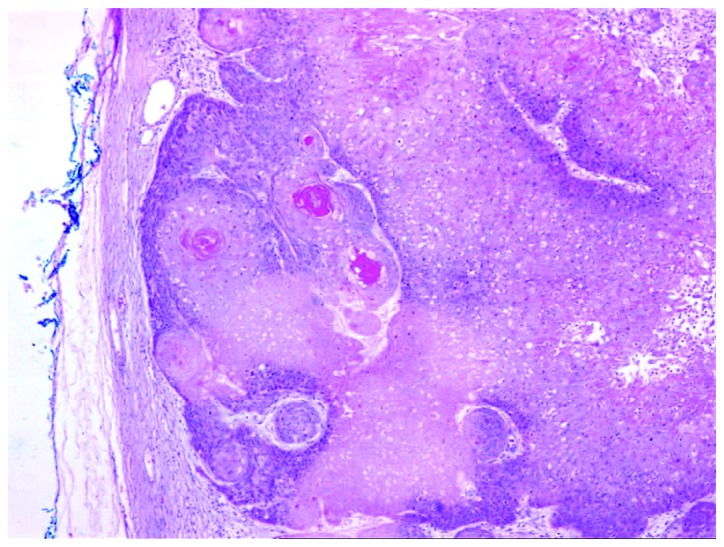
Examination of cross sections showing cells with large hyperchromatic nuclei, ghost/shadow cells and tumors observed in the keratinized regions (hematoxylin and eosin stain; magnification, ×40).

**Figure 2:**
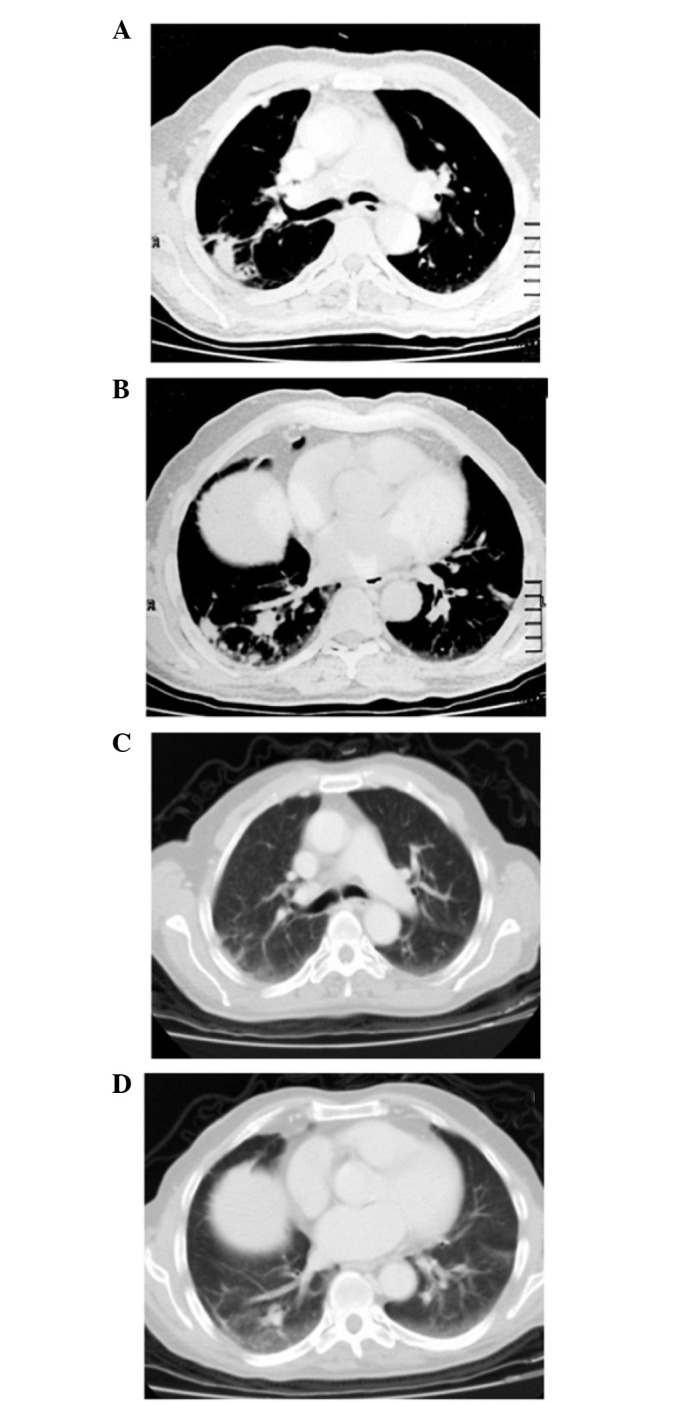
Initial chest computed tomography (CT) scans showing (A and B) multiple mediastinal lymph nodes.(at diagnosis the largest measured 12–13 mm in short-axis diameter) and multiple nodular lesions in the lung parenchyma, the largest measured 2×1.5 cm. (C and D) Follow-up chest CT scans following six courses of chemotherapy showing mediastinal lymph nodes with a short-axis diameter of <10 mm and segmental linear atelectasia in the parenchymal window, which is consistent with a complete response.

A 76-year-old male was referred to the Department of Oncology, Akdeniz University Hospital (Konyaalti, Turkey) following an excisional biopsy of a painless palpable mass located on the patient’s scalp, and the diagnosis of pilomatrix carcinoma was confirmed (Fig. 1). The physical examination revealed a surgical scar measuring ~3×3 cm in diameter on the left temporal region. Other systemic examination findings were within normal limits. A thoracic computed tomography (CT) scan showed lymph nodes with pathologically negative surgical margins in the right paratracheal mediastinal region, left paratracheal, subcarinal and bilateral hilar regions. The short-axis diameter of the largest lymph node measured 12–13 mm. The CT scan also demonstrated multiple bilateral large nodular lesions throughout the lung parenchyma, the largest of which was 2×1.5 cm (Fig. 2A and B), confirming pilomatrix carcinoma with multiple lung metastases. The patient history revealed that the patient received steroid therapy one year previously for autoimmune hemolytic anemia (AIHA). The blood and biochemical parameters of the patient were analyzed and the complete blood count, transaminase, γ-glutamyl transferase, alkaline phosphatase, blood, urea and nitrogen, and creatinine levels were found to be within the normal limits.

Following the diagnosis of metastatic pilomatrix carcinoma, the patient began combination chemotherapy with 50 mg oral cyclophosphamide (1×1) everyday and 50 mg etoposide (2×1) for five days, repeating treatment every 21 days for six courses. Following six cycles of systemic chemotherapy, a follow-up thorax CT scan showed mediastinal lymph nodes with a short-axis diameter of >10 mm and segmental linear atelectasia in the parenchymal window. The disappearance of all metastatic nodular lesions on the follow-up CT scan was evaluated as a complete response. The patient has been monitored in remission for six months.

## Discussion

Pilomatrix carcinoma is a very rare tumor with, to the best of our knowledge, ~90 cases reported in the literature. It is a skin adnexal tumor originating from hair-follicle matrix cells. While usually exhibiting locally invasive behavior, such as basal-cell skin cancer, there are numerous studies reporting patients with lung metastases. Gould *et al* (3) published the first known metastatic pilomatrix carcinoma, reporting the distant metastasis to the lungs that occurred four years following surgical excision (6,7). In the present case, pulmonary metastasis had already occurred. The patient of the present study was diagnosed with AIHA one year prior to the diagnosis of pilomatrix carcinoma, and the hemoglobin levels of the patient were within the normal range during immunosuppressive therapy. To the best of our knowledge, there is no study in the literature reporting a pilomatrix carcinoma case that was complicated by AIHA.

In the differential diagnosis, pilomatrix carcinoma should be differentiated from its more common counterparts, pilomatrixoma and basaloid cell lesions. Pilomatrix carcinoma may arise as a solitary lesion *de novo* or through transformation of a pilomatrixoma over a long period. They are slow-growing tumors of the skin, which are predominantly located in the dermis and subcutaneous fat of the neck and scalp (1). As in the present case, histopathological analysis demonstrates positive staining of pilomatrix carcinoma cells with β-catenin and epithelial membrane antigen, and hyper-proliferation and atypia observed in basaloid cells. Malignant transformation into squamous cells with translucent appearance and necrotic areas is common.

The predominant site of pilomatrix carcinoma is the head and neck, occurring in 60% of the patients that have been reported in the literature. There are also studies that have reported less common sites, including the upper extremities, torso, lower extremities and popliteal fossa (8,9). In the present case, the primary lesion was found in the scalp.

Studies in the relevant literature report tumors of various sizes. The most comprehensive data regarding tumor sizes has been provided in a study by Sau *et al* (1), in which 20 patients with tumors varying in size is reported, from 1–10 cm (mean, 4.6 cm). The tumor of the patient in the present study was 2.5 cm at its greatest diameter.

Although pilomatrix carcinoma usually demonstrate locally aggressive invasion and its metastatic potential is limited, numerous cases with metastases to the lungs, bone and lymph node have been reported. For this reason, a whole body CT scan should be performed as a diagnostic test, in order to detect all possible distant metastases. In the patient of the present study, pulmonary metastasis was detected at diagnosis. As reported in the relevant literature, the primary lesion may be a metastatic tumor despite a negative excision margin (3), which was also observed in the patient of the present study. Since this type of tumor has a strong tendency for recurrence, patient follow-up should be performed thoroughly for the timely detection of local recurrence or systemic metastasis (3).

In conclusion, there is no standard treatment protocol for pilomatrix carcinoma. While locally advanced (non-metastatic) early-stage tumors are treated by surgical resection with wide margins, various alternatives, including chemotherapy and radiation therapy, are applied to reduce the size of advanced stage tumors (4,5). A literature review revealed several cases reporting the use of various cytotoxic and immune-modulating antitumor agents (including bleomycin, 5-fluorouracil, cisplatin, vinblastine and interferon) (10). However, it is hypothesized that the complete response, which was achieved in the present case, via combination chemotherapy with oral cyclophosphamide and etoposide for lung metastases, will provide a significant contribution to the pilomatrix carcinoma literature.
